# Direct Effects of Nicotine Exposure on Murine Calvaria and Calvarial Cells

**DOI:** 10.1038/s41598-019-40796-z

**Published:** 2019-03-07

**Authors:** Emily Durham, R. Nicole Howie, Graham Warren, Amanda LaRue, James Cray

**Affiliations:** 1 Department of Oral Health Sciences, Medical University of South Carolina, 173 Ashley Avenue, Charleston, SC 29425 USA; 2 Departments of Radiation Oncology and Cell and Molecular Pharmacology and Experimental Therapeutics, Medical University of South Carolina, 173 Ashley Avenue, Charleston, SC 29425 USA; 3 Department of Pathology and Laboratory Medicine, Medical University of South Carolina, 173 Ashley Avenue, Charleston, SC 29425 USA; 4 Ralph H. Johnson Veterans Administration Medical Center, 99 Jonathan Lucas Street, Charleston, SC 29425 USA; 5 Department of Biomedical Education & Anatomy, The Ohio State University College of Medicine, 279 Hamilton Hall, 1645 Neil Ave, Columbus, Ohio 43210 USA

## Abstract

Despite the link between adverse birth outcomes due to pre- and peri-natal nicotine exposure, research suggests 11% of US women continue to smoke or use alternative nicotine products throughout pregnancy. Maternal smoking has been linked to incidence of craniofacial anomalies. We hypothesized that pre-natal nicotine exposure may directly alter craniofacial development independent of the other effects of cigarette smoking. To test this hypothesis, we administered pregnant C57BL6 mice drinking water supplemented with 0, 50, 100 or 200 μg/ml nicotine throughout pregnancy. On postnatal day 15 pups were sacrificed and skulls underwent micro-computed tomography (µCT) and histological analyses. Specific nicotinic acetylcholine receptors, α3, α7, β2, β4 were identified within the calvarial growth sites (sutures) and centers (synchondroses). Exposing murine calvarial suture derived cells and isotype cells to relevant circulating nicotine levels alone and in combination with nicotinic receptor agonist and antagonists resulted in cell specific effects. Most notably, nicotine exposure increased proliferation in calvarial cells, an effect that was modified by receptor agonist and antagonist treatment. Currently it is unclear what component(s) of cigarette smoke is causative in birth defects, however these data indicate that nicotine alone is capable of disrupting growth and development of murine calvaria.

## Introduction

Despite overwhelming data linking maternal smoking to poor fetal outcomes, an astounding 11% of women reported smoking during pregnancy^1,2^. In addition to being associated with fetal cardiovascular and musculoskeletal abnormalities, maternal smoking has been linked to incidence of craniofacial anomalies including craniosynostosis, a birth defect defined as the premature fusion of the suture(s) of the skull occurring in 1:1800–2500 births^3^. Mutations, environmental exposure, and gene/environment interactions have all been implicated as causal for instances of craniosynostosis^4^. A proposed mechanism of craniosynostosis is the disruption of the balance of proliferation and differentiation of the osteogenic precursors or stem cells in the perisutural area leading to bone overgrowth within cranial sutures^5–9^. Additionally, preservation of the intricately timed cell differentiation of the cartilaginous cranial base which contributes to calvarial growth by proper development and maintenance of the coronal ring is vital for proper craniofacial growth^10^.

Nicotine, a potent addictive stimulant in tobacco, is the primary compound in most nicotine replacement therapeutics (NRT) as well as electronic nicotine delivering products (ENDS)^11,12^. Nicotine has been linked to alteration of many physiological processes including angiogenesis^13^, cell proliferation^14^, as well as age related diseases^15^. Proper craniofacial growth and development requires a delicate balance of timed, and cell type specific cell growth, proliferation, and differentiation, and as such may be influenced by exogenous factors including maternal nicotine use^4^. It has been established that nicotine crosses the placenta during pregnancy allowing for circulation and concentration in developing fetal tissues^16^. Thus, nicotine exposure during fetal development may affect cell homeostasis within the growth sites, where calvarial growth can occur if unrestricted (calvarial sutures), and centers from which growth emanates (synchondroses), precipitating abnormal craniofacial form^17^.

Although maternal smoking is implicated in an increased risk of craniofacial abnormalities^18^, no investigations have studied if nicotine alone (apart from smoking exposure) alters calvarial development. With the advent of ENDS and NRT, it is likely that fetal exposure to nicotine will continue due to unsubstantiated safety claims. Here we investigated the direct effects of murine exposure to circulating dosages of nicotine *in utero* on craniofacial development and the effects of nicotine exposure on cell types vital to proper craniofacial growth hypothesizing that alterations will occur in a dose dependent manner.

## Results

### In utero nicotine exposure alters murine craniofacial shape

**Figure 1:**
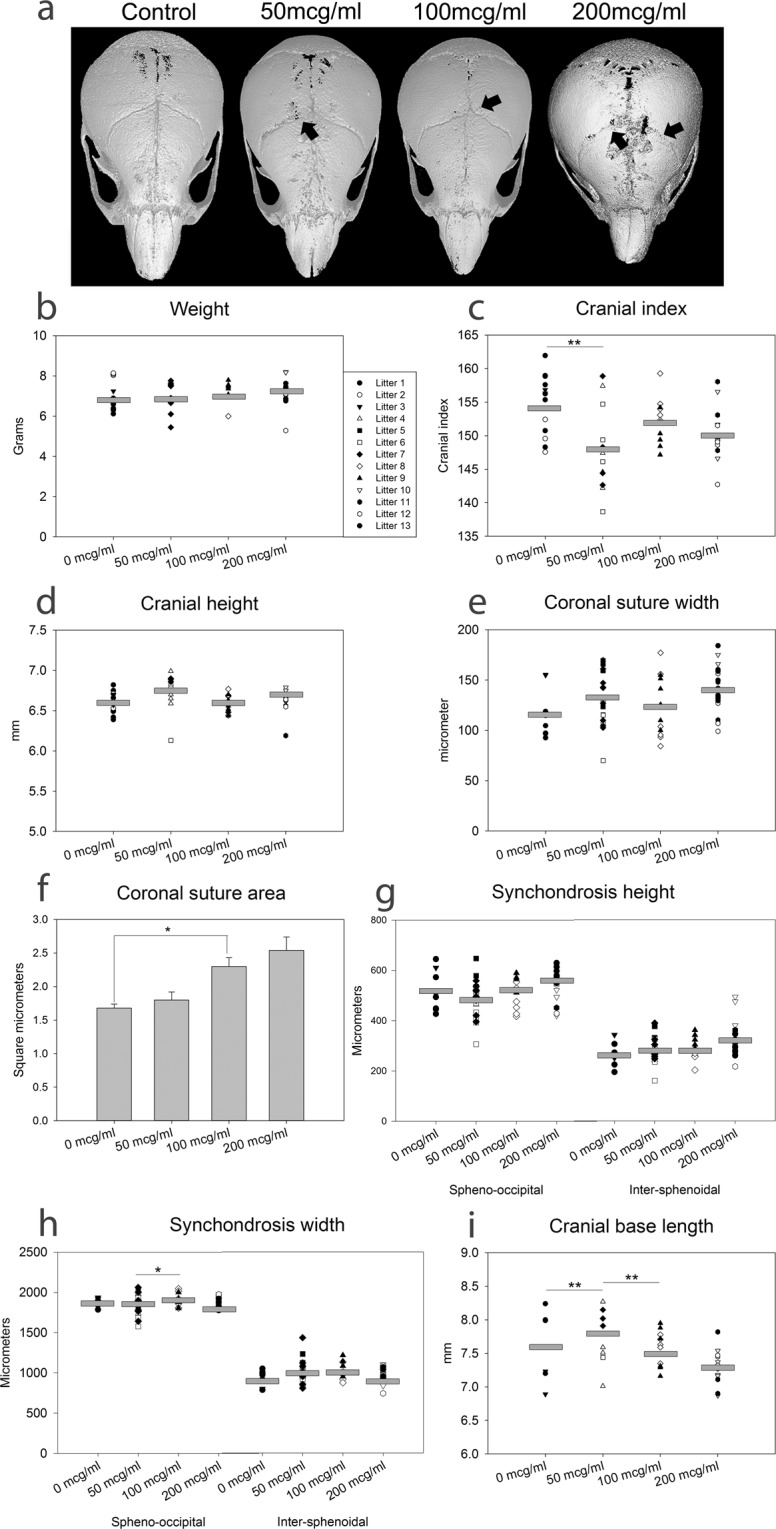
*In utero* Nicotine Exposure Alters Craniofacial Shape. (**a**) Representative 3D µCT reconstructions demonstrating dysmorphology in the high dose exposed postnatal day 15 animals. Arrows indicate potential points of suture interruption or fusion. n = 12 or 13 per exposure. (**b**) Weights of 15 day animals did not differ by sex or exposure. n = 12 or 13 per exposure, litter is indicated by symbol and grey bars indicate means. (**c**,**d**) Cranial index (cranial width × 100/cranial length) decreased in the low dose exposed however the cranial height was not affected by exposure. n = 12 or 13 per exposure, litter is indicated by symbol as noted in panel b and grey bars indicate means. (**e,f**) Coronal suture measures indicate some widening of the suture (n = 12 or 13 per exposure) and histomorphometric analysis of suture area indicates increased area with medium dose exposure. n = 4 per exposure, 2 male and 2 female from different litters. (**g,h**) The height of both the SOS and the ISS did not change with exposure, but the width of the SOS was greater in the medium dose as compared to the low dose. n = 12 or 13 per exposure, litter is indicated by symbol (**b**) and grey bars indicate means. (**i**) Low dose exposure demonstrates increased cranial base length as compared to medium dose and control. n = 12 or 13 per exposure, litter is indicated by symbol (**b**) and grey bars indicate means. *p ≤ 0.05, **p ≤ 0.01.

Representative micro-computed tomography (µCT) reconstructions from postnatal day (pn) 15 mice exposed *in utero* only to 0, 50, 100, and 200 µg/ml nicotine are included in Fig. 1a. As in clinical diagnosis of craniosynostosis, and other craniofacial abnormalities, gross dysmorphology can be noted in the high dose nicotine exposed individual. Interrupted or fused coronal suture areas can be noted along with a decrease in skull length. There was approximately equal representation of sex (27 male, 23 female), and treatment (n = 12 or 13 per treatment). No interaction was found between sex and exposure, and litter was used as a covariate for all growth assessments. Additionally, as a control for somatic measures, animal weight did not differ significantly by sex or treatment (Fig. 1b). Cranial index (cranial width x 100 / cranial length), a measure of the space occupied by the brain, is decreased in the low dose exposed individuals (p < 0.01) while cranial height remained unchanged by exposure (Fig. 1c,d). Assessment of coronal suture width indicates a trend toward increased width with exposure and a histomorphometric analysis of coronal suture area highlights an increase in area with medium dose exposure compared to control (p < 0.05) (Fig. 1e,f). The height of both the spheno-occipital (SOS) and the inter-sphenoidal synchondroses (ISS) indicated no change due to nicotine exposure however, the width of the SOS of the cranial base vital for proper growth increased with medium dose exposure as compared to low dose (p < 0.01) (Fig. 1g,h). Investigation of the cranial base region indicates an increase in length with low dose exposure compared to medium dose and control exposures (p < 0.01 and p < 0.05 respectively) (Fig. 1i).

### Analysis of Target Nicotinic Receptors

**Figure 2:**
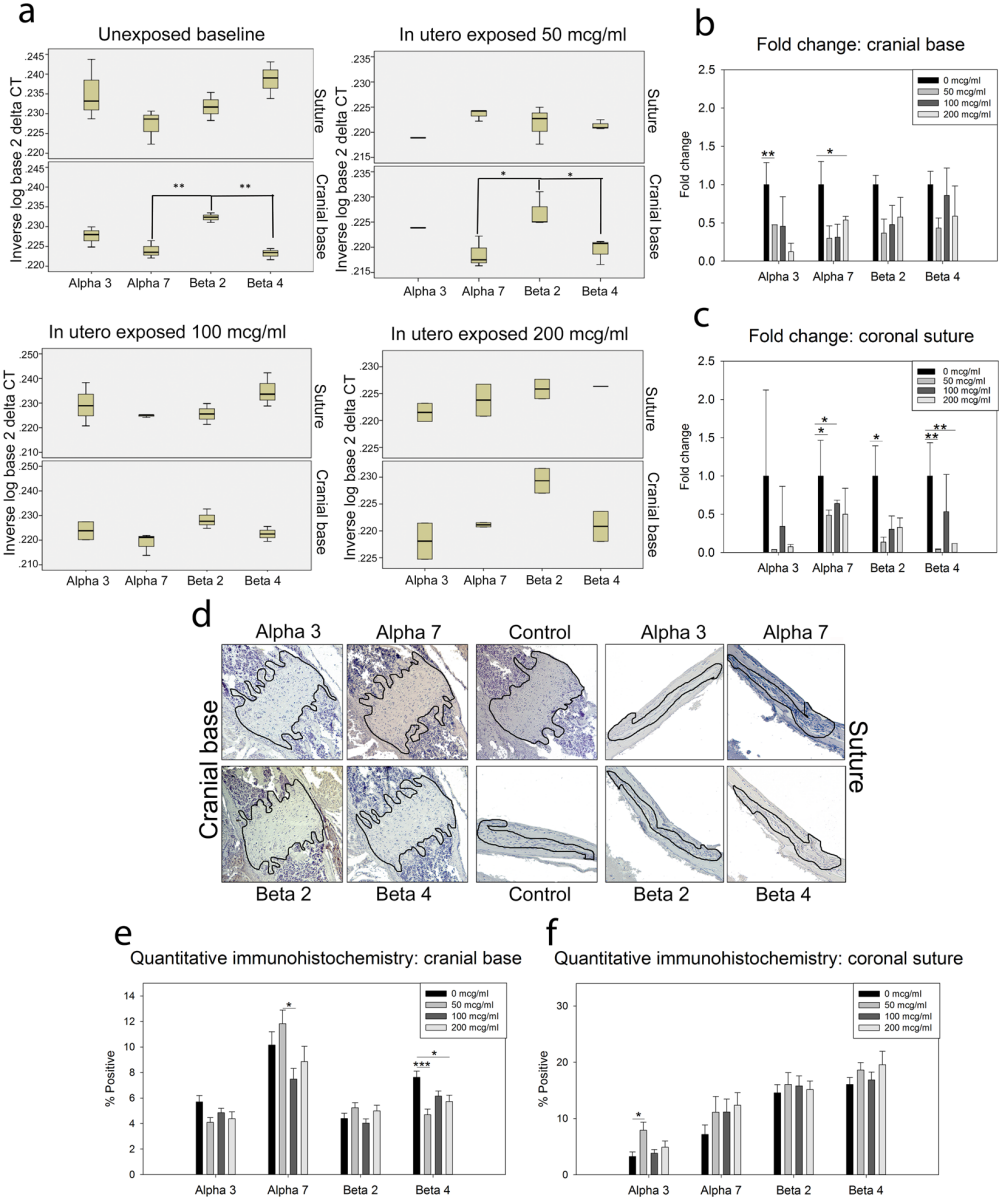
Analysis of Target Nicotinic Receptors. (**a**) qrt-PCR analysis for target nicotinic receptors on murine suture and synchondrosis RNA isolated from postnatal day 15 pups after nicotine exposure. Gene expression for nicotinic receptors may be modulated with exposure. n = 3 per group. (**b**,**c**) Fold change in nicotinic receptor expression compared to control (no exposure) in tissue from the cranial base (**b**) and coronal suture (**c**) regions. (**d**) Representative immunohistochemical staining for target receptors identified in tissues of interest (suture and cranial base) in high dose exposed and control (Secondary Antibody Only). (**e**,**f**) Quantification of percent staining of each target. n = 4 per exposure, 2 males and 2 females from different litters. Data presented as mean ± standard error of the mean. *p < 0.05, ***p < 0.001.

PCR was used to determine the presence of nicotinic receptors within the tissues of interest: coronal suture and cranial base. A comprehensive investigation revealed four receptor isoforms to be expressed within those tissues (Table 1). Further investigation of these target receptors, (Chrna3, Chrna7, Chrnb2, Chrnb4) revealed some modulation of gene target expression with exposure in cranial base tissue (Fig. 2a). Nicotine exposure resulted in a significant down regulation of Chrna3 (low dose compared to control p = 0.003) and Chrna7 (high dose compared to control (p = 0.0131) in tissue isolated from the cranial base (Fig. 2b). In the coronal suture derived tissue, nicotine exposure also caused a down regulation of Chrna7 (low dose (p = 0.0387) and medium dose (p = 0.0241) compared to control). Additionally, nicotine exposure downregulated both Chrnb2 (low dose compared to control p = 0.05) and Chrnb4 (low dose (p = 0.002) and high dose (p = 0.002) compared to control) (Fig. 2c). Immunohistochemical identification of all four nicotinic receptors within the tissues of interest was successful (Fig. 2c) and quantification of percent positive staining in cranial base tissue indicated a decrease in nicotinic acetylcholine receptor α7 between low and medium doses (p < 0.05), and β4 between control and low (p < 0.001) and control and high dose exposures (p < 0.05). For the coronal suture tissue, only an increase in positive staining for nicotinic acetylcholine receptor α3 was found in the low dose exposed as compared to control (p < 0.05) (Fig. 2e,f-).

**Table 1:** PCR Identification of Nicotinic Receptors.

Receptor	Forward	Reverse	Size	Suture	Cranial Base	Expression only after Nicotine Exposure	No Expression in Either Tissue	TaqMan Gene Expression Assay	qrtPCR <40 cyc
Chrna1	TAA CCC GGA AAG TGA CCA GC	TGC AAT GTA CTT CAC GCC CT	676			×		Mm00431629_m1	
Chrna2	AAA GTC ACG CTT GCA GAC TC	GAT GTT GCC AAA CTC AGC CG	419				×		
Chrna3	CGC CTG GTC TCA CAC TCA TT	CTG CCG AAG TCC ACA CAT CT	577	×	×			Mm00520145_m1	×
Chrna4	CCT CGT CTA GAG CCC GTT C	TTC AGA TGG GAT GCG GAT GG	381				×	Mm00516561_m1	
Chrna5	GAT CTC GAA TGC AGG GTT GTT GC	CAG AGA GAC CAG CAC GGA AG	720				×	Mm00616329_m1	
Chrna6	CTG CCC AAT GGA CAT CAC CT	ACC CAC TTG GGC ATG GTA TG	552	×	×			Mm00517529_m1	
Chrna7	CCT GCT CCC CAA CAC ATG AT	GCC GGT GAT GGG TGT AAG AA	473	×	×			Mm01312230_m1	×
Chrna9	AGC TGC GTC TCC AGT CAT TC	TGC TGT CTC TAC GGC TTT GA	355				×		
Chrna10	AGT CAT ATG GAA AGG GAC GGA A	TGG AAA CCA GAG ATT GCG GC	141	×	×			Mm01274155_m1	
Chrnb1	TTC TAC CTC CCA CCA GAT GC	GGT ATG GAG GGA GCT TGT GA	274	×	×			Mm00680412_m1	
Chrnb2	CAA TGC TGA CGG CAT GTA CG	CTA CGC AGG GGA TGA TGA GG	377	×	×			Mm00515323_m1	×
Chrnb3x1	CAG GCT TCC TAC GGG TCT TC	ATT CCT GCT TCA GCC ACA CG	267		×			Mm00532602_m1	
Chrnb3x2	CGA GGC TCT GAA CAA CTT GT	TGG TCT GTC CAT TCC ACA TCT	356	×					
Chrnb4	CTC ACT CGC GGT TCC ATT GT	ATA GCC AGC GAC GAC GTG ATG AG	797	×				Mm00804952_m1	×
Chrnd	GTG GGA GAT AGT GCA TCG GG	CAT GCC GCT CTG ATT GCT TC	596			×		Mm00445545_m1	
Chrne	TGG CCT ACG ACA GCA ATG TT	CTG CGG TCC AAG TTC CGT	858				×		
Chrng	AGA GAC CTC AGC TCC TCT TGC	TCC ACA GGC CTT CGT AGT CT	282	×	×			Mm00437419_m1

### Primary Cell Treatment with Nicotine, Nicotinic Receptor Agonist, and Antagonists

**Figure 3:**
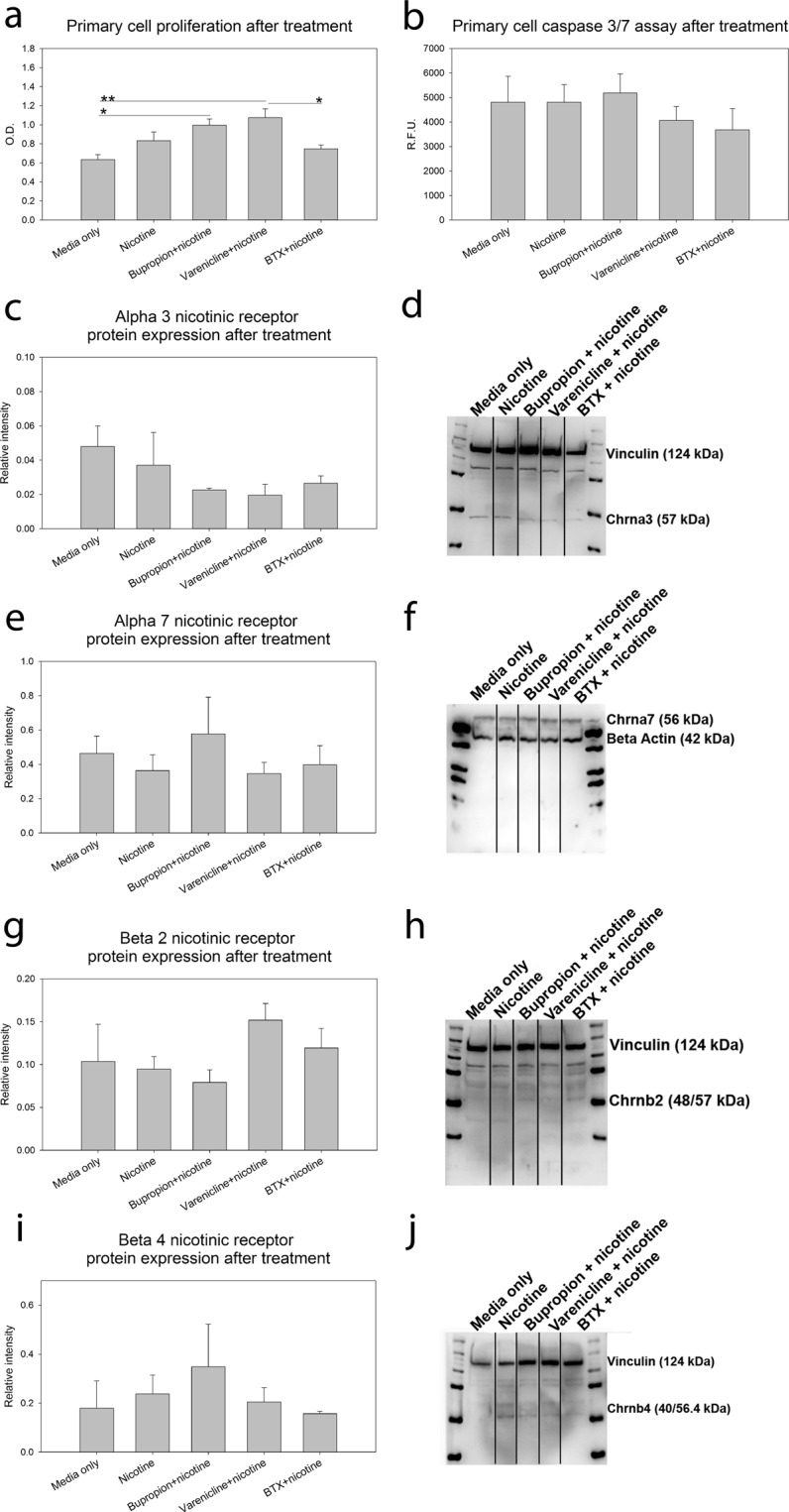
Primary Cell Treatment with Nicotine, Nicotinic Receptor Agonist, and Antagonists. (**a**) Primary murine coronal suture cell proliferation increased with treatment with nicotinic receptor agonist Varenicline in combination with nicotine over both control and treatment with nicotine in combination with antagonists. Further, proliferation increased with combined treatment of specific antagonist Bupropion with nicotine over control. n = 7. (**b**) Apoptotic activity was not affected by treatment with nicotine, or nicotine in combination with agonist or antagonists. n = 7. (**c**–**j**) Target nicotinic receptor presence was not affected by treatment with nicotine alone or in combination with agonist or antagonists as determined by western blot. Western lanes divided to indicate removal of intervening lanes with agonist and antagonist treatment alone. See Supplementary Fig. 1 for full blots. n = 4. Data presented as mean ± standard error of the mean. *p < 0.05, **p < 0.01.

In order to determine if cells making up the growth sites and centers of the skull could be specifically affected by nicotine exposure to precipitate the noted craniofacial abnormalities primary murine coronal suture cells were treated with nicotine in the presence of a nicotinic receptor agonist and antagonists. Nicotinic receptor agonist Varenicline combined with nicotine increased proliferation as compared to control cells treated with only proliferation media (p < 0.001). Nicotinic receptor antagonist Bupropion in combination with nicotine also increased proliferation above media only control (p = 0.012). Interestingly, treatment with nicotinic receptor antagonist α-bungarotoxin (BTX) decreased cell proliferation compared with combined nicotine and Varenicline treatment (p = 0.03) (Fig. 3a). An investigation into the effect of these treatments on cell apoptotic activity indicated no apoptotic response to these treatments over baseline media only control (Fig. 3b). Assessment of nicotinic receptor protein expression for the nicotinic acetylcholine receptors of interest (α3, α7, β2, β4) indicated no significant change in protein in primary coronal suture derived cells treated with nicotine alone or in combination with the agonist (Varenicline) or antagonists (Bupropion and BTX) (Fig. 3c–f, Supplementary Fig. 1).

### Isotype Cell Treatment with Nicotine, Nicotinic Receptor Agonist, and Antagonists

**Figure 4:**
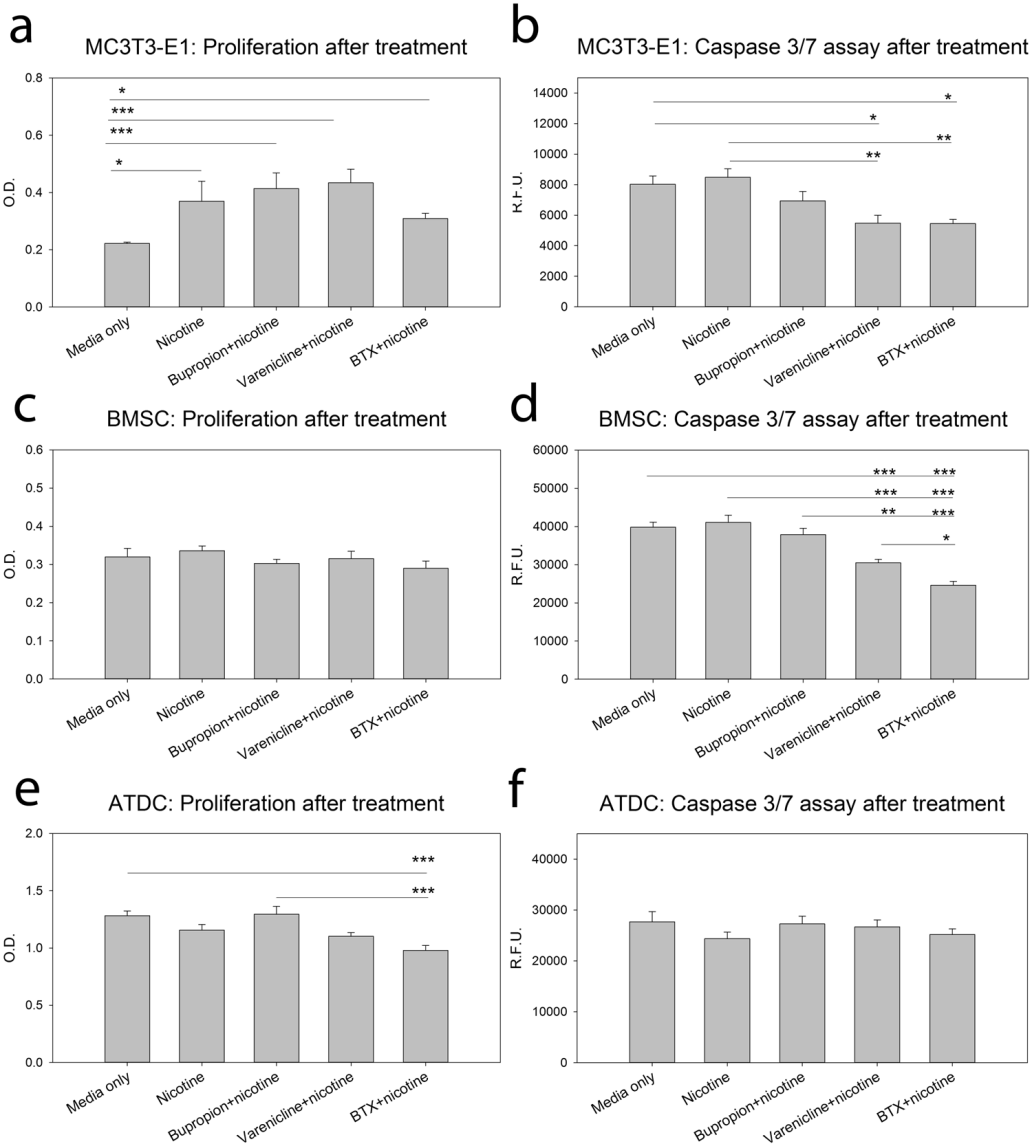
Isotype Cell Treatment with Nicotine, Nicotinic Receptor Agonist, and Antagonist. **(a**,**b**) Calvarial derived pre-osteoblasts (MCT3T-E1) cells increase in proliferation with nicotine treatment alone or in combination with nicotinic receptor agonist or antagonists as compared to control. Further, treatment with nicotinic receptor agonist (Varenicline) and antagonist (BTX) combined with nicotine decreases apoptotic activity in these cells compared to control whereas, nicotine in isolation increases apoptotic activity compared to combined treatments. n = 3. (**c**,**d**) Murine Bone Marrow Stromal Cells showed no proliferative response to treatments, however treatment with BTX with nicotine decreased apoptotic activity as compared to all other treatments. Further, treatment with nicotinic receptor agonist Varenicline with nicotine also reduced apoptotic activity compared to control and nicotine alone treatments. n = 3 (**e**,**f**) ATDC chondrocyte cells reduce proliferation with BTX plus nicotine treatment as compared to control and nicotine with Bupropion. These cells showed no effect on apoptotic activity with treatment. n = 3 Data presented as mean ± standard error of the mean. *p < 0.05, **p < 0.01, ***p < 0.001.

Since the suture mesenchyme and cranial base are made up of multiple cell types *in situ*, we exposed isotype cells pre-osteoblasts (MC3T3-E1), bone marrow derived stromal cells (BMSC), and chondrogenic cells (ATDC) to the same treatments as above to determine if the observed effects were cell type specific. Treatment with nicotine alone or in combination with receptor agonist or antagonists increased proliferation over control for pre-osteoblast cells (p < 0.05) (Fig. 4a). Treatment of pre-osteoblast cells with nicotine in combination with Varenicline and in combination with BTX decreased apoptotic activity compared to control media only treatment (p = 0.012 and p = 0.011 respectively) and compared to nicotine alone treatment (p = 0.002) (Fig. 4b). BMSC cells did not respond to treatments with a change in proliferation, however nicotine with BTX treatment decreased apoptotic activity as compared to all other treatments (p < 0.05) (Fig. 4c). Treatment with nicotine and Varenicline also decreased apoptotic activity as compared to all other treatments save the nicotine and BTX treatment (p < 0.05) (Fig. 4d). Treatment with nicotine and BTX also decreased proliferation in ATDC cells as compared to control and nicotine with Bupropion treatment (p < 0.001). No changes in apoptotic activity due to treatment were observed in ATDC cells (Fig. 4e,f).

## Discussion

Here we sought to model recurrent nicotine exposure as in cigarette smoking and use of ENDS in a murine model to determine if *in utero* exposure to nicotine could alter calvarial development. We observed altered suture morphology on gross analysis of µCT images; however, these suture specific alterations were not corroborated by our more in-depth analyses. Observation of postnatal day 15 mouse pups indicated that nicotine could alter craniofacial form reducing the cranial index and affecting the growth trajectory of the cranial base. Though we did not observed suture fusion as predicted by the correlation between maternal smoking and increased risk of craniosynostosis, our data support this association as evidenced by the noted altered craniofacial form with nicotine exposure alone^18^. Interestingly, we did not observe a reduction in weight of exposed animals at the 15 day time-point indicating that if there was a reduction in birth weight associated with *in utero* nicotine exposure as in the human population, it was regained quickly after birth in this murine model regardless of long term effects of *in utero* nicotine exposure^18,19^.

Though we did not observe the expected additive dose response to nicotine exposure, we did see aberrant craniofacial shape at each exposure indicating that each dose has an effect and that there may be redundancies allowing for compensation for teratogenic insult. Exposure to medium dose nicotine resulted in a wider synchondrosis and greater coronal suture area. Low dose exposure decreased cranial index and cranial base length. Abnormal cranial base, or calvarial suture growth can give rise to aberrant craniofacial shape^20^. Changes in the growth trajectory of the cranial base, which is comprised of growth centers (synchondroses) associated with maintenance of the coronal ring, can contribute to craniofacial growth disturbances that compound throughout growth resulting in distinct abnormalities such as premature suture fusion^21,22^. It is also possible that individual sutures are differentially affected by nicotine exposure, or that compensatory growth occurring prior to 15 days obscures the effects of nicotine. Thus, the different responses to different dosages may result in dose specific phenotypes later in development.

In addition to the gross morphological changes observed, our site-specific interrogation of the 17 different nicotinic acetylcholine receptors revealed that only 4 receptors were robustly expressed in the tissues most implicated in craniofacial development: suture and synchondrosis. Importantly, this is the first time these receptors have been identified within these tissues and their presence indicates that nicotine can affect specific cells within these tightly regulated spaces. Chrna3, Chrna7, Chrnb2, and Chrnb4 expression was variable across doses of nicotine displaying perhaps that exposure to nicotine can cause receptor internalization and functional downregulation long term, even when exposure has been removed. Nicotine exposure has been shown to increase presence of nicotinic acetylcholine receptors at the cell surface in a process termed upregulation which is specific to these receptors. This process does not however occur by increasing mRNA levels which suggests that posttranslational conformational changes may be more important to the functional capabilities of these receptors^23^. Further, though nicotine use is associated with a dose dependent increase in neurologic nicotinic acetylcholine receptors, receptor levels in the brain of individuals who have ceased smoking return to levels similar to or below those individuals who have never directly consumed nicotine. Therefore, our observed down regulation of nicotinic acetylcholine receptors may indicate this eventual reduction in receptor presence with removal of nicotine exposure^24^. The variation of receptor expression across nicotine doses, in conjunction with the presence of the receptors outside of the nervous system, provide further evidence that the physiological role of these receptors is not yet fully understood^23^.

The nicotinic acetylcholine receptor α7 is known to act as a homopentamer complex and rapidly desensitizes allowing for greater activity compared to other receptors. Interestingly, the other nicotinic acetylcholine receptors identified in the tissues of interest are known to act in a heteromeric complex between α3 and either β4 (primarily) or β2 (potentially). The ratio of the presence of the α and β receptors can dramatically affect their function indicating that in these tissues, as in the lung, the α7 receptor may be the primary mediator of the effects of nicotine^23,25^. Nicotine acts through these receptors by regulating diverse signaling pathways including cell proliferation and death^26,27^. These data confirmed that nicotine could have a site-specific and dose-specific effect.

Both the growth sites (sutures) and centers (synchondroses) require a tightly controlled balance of cell proliferation, differentiation, and death to precipitate proper craniofacial development and form. In most instances of craniosynostosis, and other craniofacial abnormalities the precise mechanism by which abnormal growth occurs is elusive however, the coronal suture along with the synchondroses of the cranial base as part of the coronal ring have been implicated in the pathogenesis of craniosynostotic phenotypes^4,28^. One theorized mechanism for craniosynostosis is a disruption of the balance of cells within these tightly controlled areas. Our specific interrogation of the effects of nicotine exposure in isolation and in combination with nicotinic receptor agonist (Varenicline) and antagonists (Bupropion and BTX) provided mixed results depending on the cell type. In the heterogeneous primary suture derived cells, nicotine in combination with pan-nicotinic acetylcholine receptor antagonist (Bupropion) increased proliferation as did nicotine in combination with the agonist (Varenicline). These pharmacological agents compete with nicotine for receptor binding and have been known to preferentially induce expression of one nicotinic acetylcholine receptor conformation over another in addition to altering trafficking and expression of receptors^29^. The α7 specific antagonist (BTX) restored proliferation to control levels indicating that in these cells, modulation of this receptor specifically can ameliorate the response to nicotine. Using both antagonists and an agonist in combination with nicotine allowed for an assessment of the downstream effects of nicotine exposure. The specific antagonistic response observed when nicotine was used in combination with the α7 specific antagonist (BTX), indicates that the effects of nicotine in these cells may be specific to that receptor which may therefore be a viable therapeutic target. Because we precipitated a smaller than expected response in the heterogeneous primary cells, our investigation became more focused towards the specific cell type affected.

In order to determine if the effects of nicotine observed in the heterogeneous primary cell populations were cell type specific, we treated isotype cells that should be present in the heterogeneous population with nicotine and nicotinic receptor agonist and antagonist. Incubation with nicotine and receptor agonist, and antagonists allowed for an assessment of which receptors present may be modulated by nicotine exposure. Bupropion, which has affinities for multiple nicotinic receptors, did not consistently appear to modulate proliferation across the cell types. However, nicotine plus BTX, which is specific to nicotinic acetylcholine receptor α7 was observed to decrease proliferation levels approaching control levels for all cells, except the BMSCs. These data suggest potential cell specific effects and indicate that the effects of nicotine observed here may be α7 specific and thus highlighting this receptor as a potential specific therapeutic target. As ablation of stem cells, and disruption of the balance of proliferation and differentiation within the suture space are proposed mechanisms of suture fusion, nicotine is a potential direct causative agent for abnormal craniofacial growth and development^9^. It remains to be determined what other components of nicotine use increase the effects observed here within the context of craniofacial birth defects. Further, our observation that the nicotinic receptor agonist affects cell proliferation and death indicates that these drugs often used for smoking cessation during pregnancy may have detrimental effects on fetal development as well.

Even though nicotine exposure was not specifically metered, these results clearly indicate that *in utero* exposure to nicotine is capable of altering calvarial and craniofacial growth and development. We have positively identified nicotinic acetylcholine receptors that reside within the craniofacial growth sites and centers allowing for site specific effects. *In vitro* use of nicotinic receptor agonist and antagonists indicated that the effects of nicotine in these areas of interests may be nicotinic acetylcholine receptor α7 specific. Further, nicotine has cell type specific effects disrupting cell proliferation in cells resident to craniofacial growth sites and centers that are vital for proper growth and development. Though quitting smoking during pregnancy is the gold standard, we provide some evidence here that nicotine in cessation treatments and other nicotine delivery systems may negatively affect fetal development. As new nicotine delivery technologies (i.e. inhalers, e-cigarettes) and new cessation therapeutics focusing on controlled nicotine delivery become more popular the effect that nicotine has on the developing skull has the potential to be the next public health crisis in birth defects research.

## Methods

### Animal Model *in vivo* Exposure

To mimic the effects of recurrent nicotine exposure to the fetus as in maternal smoking and other nicotine related exposures, adult wild type, C57BL6 (*Mus musculus*, Jackson Laboratories, Bar Harbor, ME) male and female mice were utilized to produce *in utero* nicotine exposed litters. Nicotine (Sigma Aldrich N3876, St. Louis, MO) was diluted in drinking water at 0, 50, 100, and 200 µg/ml throughout pregnancy^25,30–34^. Based on an average daily intake of 4 ml of water, our scaled dose ranged between 200 and 800 µg / day while the range of nicotine intake for active smokers is between 10 and 100 mg / day and those using alternative nicotine delivery systems experience an even wider range of exposure^35,36^. Based upon historic breeding colony metrics, we paired male and female mice for 7 days with over 80% of parings resulting in pregnancy within the first 48 hours. After 7 days, males were removed to other pairings, or individual cages. Females continued nicotine treatment until birth of the litters at ~E20. Twelve or 13 mouse pups from two to four litters per exposure were grown to 15 days postnatal (pn) (the earliest time point where craniofacial abnormalities have been observed in previous teratogenic studies^10,37^) when they were sacrificed and skulls were fixed with 4% paraformaldehyde, then switched to 70% Ethanol for micro-computed tomography (µCT) analysis, and finally processed for paraffin based histology. Animal use protocols were approved by the Medical University of South Carolina Institutional Animal Care and Use Committee (AR#3403). All breeding procedures were carried out in an Association for Assessment and Accreditation of Laboratory Animal Care International accredited facility where all husbandry and related services are provided by the Division of Laboratory Animal Resources. All procedures and the reporting thereof are in compliance with the Animal Research: Reporting *in Vivo* Experiments (ARRIVE) guidelines^38^.

### Micro-computed Tomography (µCT) and Radiographic Analyses

µCT images were obtained on mouse pup skulls with a SkyScan 1174 (Kontich, Belgium) at a 22.57 µm voxel resolution. Scans were obtained on 50 animals (27 male; 23 female). Mouse skulls were reconstructed with CTVox software v2.3.0 r810 (Skyscan). Threshold settings were then set to only visualize bone volume within the skull. Measurements of the widths, and heights (thickness of the bones of the cranial base) of the cartilaginous regions between ossified centers, spheno-occipital (SOS) and inter-sphenoidal (ISS) synchondroses at the midline of the cartilaginous segment were recorded per published methodology^10^. Additionally, the width of the coronal suture was measured per published methodology at 25, 50, and 75 percent of its length^37^.

After µCT, dorsoventral radiographs were obtained using a faxitron X-Ray instrument and PPL film (Carestream, NY, USA). Skulls were then bisected along the sagittal suture and lateral radiographs were also obtained. From these radiographs, skull length (parietal bone to nasion), width (at the widest portion of the calvarium) and height (from opisthion to the frontal-parietal suture) were assessed. From these measures, cranial index (cranial width x 100 / cranial length), a measure of the space occupied by the brain was also assessed.

### Hematoxylin and Eosin Suture Histomorphometry

Four representative samples (2 males and 2 females from separate litters) per group (control = no dose, low dose = 50 µg/ml, medium dose = 100 µg/ml, high dose = 200 µg/ml) were decalcified in 0.25 M EDTA at pH 7.4 for 10 days with changes every 3 days. Skulls were then dehydrated in a graded series of ethyl alcohol (70–100%), cleared in xylene, and embedded in paraffin. Prior to embedding, the calvaria was removed from the cranial base and was bisected along the sagittal suture to facilitate cutting through the coronal suture. The remaining cranial base was also bisected and embedded to facilitate cutting through the cranial base synchondroses coronally. All tissues were sectioned at 8 µm using a rotary microtome prior to mounting on Super Frost Plus (ThermoFisher Scientific, Waltham, MA) slides. Hematoxylin and eosin staining proceeded by standard protocol. Stained sections were photographed using a Motic Inverted Microscope with attached camera (Motic, British Columbia Canada) and measured using Image J Software (National Institutes of Health)^37,39^.

### Tissue Based Qualitative and Quantitative Polymerase Chain Reaction

A selected set of skulls (n = 3 per exposure from at least two litters) were not fixed but placed in ice cold RNAlater (ThermoFisher Scientific). Subsequently, the cranial base including the SOS and ISS and intervening bony tissue, and the coronal suture were identified and separately isolated. The extirpated tissue was then homogenized in a liquid nitrogen cooled mortar and digested in TRIZOL (ThermoFisher). RNA was then isolated using the Qiagen RNEasy mini kit (Qiagen, Valenica, CA, USA) according to manufacturer’s protocol. Quantity and quality of RNA was assessed using a Synergy H1 Microplate reader and a Take3 Microvolume Plate (BioTek, Winooski, VT, USA). Complimentary DNA Synthesis was performed using Superscript II Reverse Transcriptase and random hexamer primer following manufacturers protocol (ThermoFisher Scientific). Presence of the nicotinic acetylcholine receptors was determined via PCR using cDNA, designed primers from Integrated DNA Technologies (Coralville, IA) (Table 1), Platinum Taq DNA Polymerase (ThermoFisher Scientific), and separation on 2% agarose gels employing beta actin as a control. Once the list of target receptors was narrowed, the cDNA was subjected to quantitative PCR using Applied Biosystems TaqMan Gene Expression Master Mix and targeted TaqMan gene expression assays for: Chrna3, Chrna7, Chrnb2, Chrnb4. Data were normalized to 18 S (Mm03928990_g1) ribosomal RNA expression by ΔCT. Quantitative data were compared for gene expression change due to treatment by ΔΔCT methodology. We used statistical analyses for qrt-PCR data as previously published to determine statistical differences for gene expression after nicotine exposure for targets of interest^40^. Differences were considered significant if p ≤ 0.05. Data are presented as inverse of the log base 2 delta CT to allow for direct visual comparison between targets and morphological sites (Fig. 2a), and by fold change compared to control no exposure top highlight changes due to exposure (Fig. 2b,c).

### Immunohistochemistry

For immunohistochemistry, representative samples (n = 4, 2 males and 2 females from separate litters) from each group were blocked with 3% hydrogen peroxide and then washed 3 times in phosphate buffered saline and blocked in 1% goat serum or donkey serum with 1% bovine serum albumin. Sections were incubated with the following primary antibodies overnight at 4 degrees: nicotinic acetylcholine receptor α3 (AbCam Cambridge, MA, ab183097, 1:50), α7 (ab10096, 1:200), β2 (ab129276, 1:100), β4 (ab189174, 1:400). Then, sections were washed 3 times in phosphate buffered saline and incubated with HRP conjugated secondary antibody for 1-hour (ab6721, ab6885, 1:250) and diaminobenzidine (DAB) (Vector Laboratories, Bulingame, CA) chromogen was used according to manufacturer’s protocol to identify immunoreactive structures. Coronal suture and whole synchondroses including abutting trabecular bone were digitally isolated for direct comparison between control and nicotine exposed individuals (outlined in Fig. 2d). At least 3 sections 30 μm apart per individual per treatment for each target were analyzed using Image J Software and the IHC Profiler Open Source Plugin for automated scoring of percent positivity^41^.

### Cell culture, Treatment, and Assays

To determine the specific effect of nicotine on the cells comprising the calvarial growth sites and centers, primary, wild type coronal suture cells isolated as previously described^42^, murine bone marrow stem cells (BMSC)^43^, as well as isotype cell lines pre-osteoblasts MC3T3-E1 and chondrogenic cells ATDC5 (ATCC, USA), were cultured at 37 °C in a humidified 5% CO_2_ incubator. MC3T3-E1 cells were cultured in αMEM (Lonza, USA), while the remaining cells were cultured in DMEM, supplemented with 10% FBS, 1% penstrep, and 0.2% amphotericin with media changes twice per week until 95% confluence was reached. At the time of confluence, cells were seeded at a density of 4,000 cells per well for cell proliferation and apoptosis assays. Primary coronal suture cells were also seeded at a density of 300,000 cells/well for whole cell protein collection. After seeding, cells were treated with proliferation media (control), nicotine 25 ng/ml alone (mimicking the concentration in blood of an active smoker^35,36^), or in combination with nicotine receptor agonist (Varenicline 20 ng/ml; ToCris #3754, Avonmouth, Bristol, United Kingdom; trade name CHANTIX) or antagonists (Buproprion Hydrochloride 100 ng/m; ToCris #2831; Trade name WELLBUTRIN), or α-Bungarotoxin (BTX) 100 ng/ml (ToCris #2133) for 7 days with media changes every 2–3 days^23^.

Cell viability (proliferation) was assessed with the colorimetric MTS assay and apoptosis was assessed using the APO-ONE Caspase3/7 Assay (Promega, USA) per manufacturers’ protocol using a Gen5 plate reader (BioTek, Winooski, VT, USA).

### Western Blots on Protein from Primary Coronal Suture Cells

Protein from primary cells (n = 4 isolations of primary cells, 2 male and 2 female from different litters) was extracted with cold RIPA buffer (ThermoFisher). Total protein was quantified using a Bradford assay (ThermoFisher). Protein extracts were separated by 10% SDS-PAGE. Equal amounts of protein per lane were loaded and transferred onto PVDF membrane (BioRad, Hercules, CA, USA). The blots were probed with the following antibodies diluted in Tris-buffered saline, 0.1% Tween 20 with 5% (wt/vol) bovine serum albumin: anti-nicotinic acetylcholine receptor α3 (ab183097, 1:400), α7 (ab10096, 1:300), β2 (ab189174, 1:250), β4 (ab129276, 1:250), Anti-Vinculin (ab129002, 1:480,000), beta actin (Cell Signaling, 4967 S, 1:10,000). Incubation with HRP conjugated anti-rabbit (ab6721; 1:3000) or anti-goat IgG (ab189174, 1:3000) followed. The protein was visualized by enhanced chemiluminescence ECL Clarity (BioRad) detection reagents. Band intensity was quantified using NIH Image J software.

### Statistical Analyses

Previous pharmacological studies in our laboratory suggested an n = 12 per group to achieve sufficient power for our *in vivo* measures (α = 0.05, β = 0.80, r >0.40)^44^. Growth measures were screened for normality and homogeneity of variance and subjected to Analysis of Covariance to allow for incorporation of litter as a covariate^45^ while comparing effects by dose; p ≤ 0.05 was considered significant for post-hoc Bonferonni analyses where appropriate. Further, a two-way ANOVA was implemented for all growth measures to determine if there were any significant interaction terms by sex. All statistical analyses were completed using SPSS 23.0 (IBM, Armonk, NY, USA). Data are categorized by litter with the mean for each exposure identified using a grey bar or are represented as mean ± standard error of the mean.

## Supplementary information

Supplementary Data

## Data Availability

Materials, data and associated protocols available upon request to corresponding author James Cray Jr., Ph.D., Associate Professor 843–792–6940 james.cray@osumc.edu.
