# Supplementary material for: Direct Effects of Nicotine Exposure on Murine Calvaria and Calvarial Cells
Source: Sci Rep. 2019 Mar 7;9:3805. doi: 10.1038/s41598-019-40796-z (PMC6405741; doi:10.1038/s41598-019-40796-z)
Supplement: Supplementary file 1 — Supplementary Data [file 41598_2019_40796_MOESM1_ESM.pdf]

Title: Direct Effects of Nicotine Exposure on Murine Calvaria and Calvarial Cells

Authors: Emily Durham[a] (durhame@musc.edu), R. Nicole Howie[a] (rnhowie@gamil.com), Graham Warren[b] (warrengw@musc.edu), Amanda LaRue[c,d] (laruerc@musc.edu), James Cray[e,f] (James.Cray@osumc.edu)*

Supplementary Information

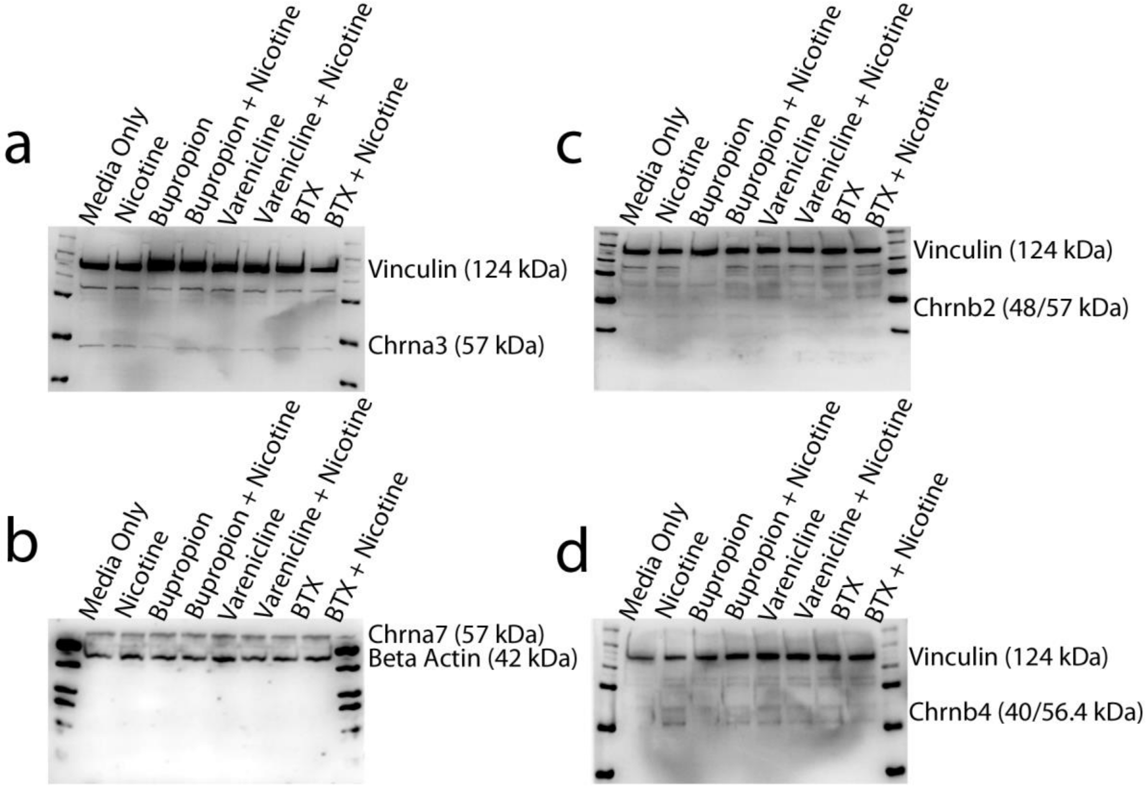

Supplemental Figure 1: Whole Western Blots Primary Cell Treatment with Nicotine, Nicotine Receptor Agonist, and Antagonists. Target nicotinic receptor Alpha 3 (a), Alpha 7 (b), Beta 2 (c), and Beta 4 (d) presence was not affected by treatment with nicotine, agonist, or antagonist alone or in combination as determined by western blot. N=4.
